# Reduction of wing area affects estimated stress in the primary flight muscles of chickens

**DOI:** 10.1098/rsos.230817

**Published:** 2023-11-29

**Authors:** Grace A. T. Hong, Bret W. Tobalske, Nienke van Staaveren, Emily M. Leishman, Tina M. Widowski, Donald R. Powers, Alexandra Harlander-Matauschek

**Affiliations:** ^1^ Campbell Centre for the Study of Animal Welfare, Department of Animal Biosciences, University of Guelph, 50 Stone Road E, Guelph, Ontario Canada, N1G 2W1; ^2^ Centre for Genetic Improvement of Livestock, University of Guelph, 50 Stone Road E, Guelph, Ontario Canada, N1G 2W1; ^3^ Centre for Nutrition Modelling, Department of Animal Biosciences, University of Guelph, 50 Stone Road E, Guelph, Ontario Canada, N1G 2W1; ^4^ Division of Biological Sciences, University of Montana, 32 Campus Drive, Missoula, MT 59812, USA; ^5^ Department of Biology, George Fox University, 414N Meridian St, Newberg, OR 97132, USA

**Keywords:** laying hen, estimated muscle stress, physiological cross-sectional area, feather loss, keel bone

## Abstract

In flying birds, the pectoralis (PECT) and supracoracoideus (SUPRA) generate most of the power required for flight, while the wing feathers create the aerodynamic forces. However, in domestic laying hens, little is known about the architectural properties of these muscles and the forces the wings produce. As housing space increases for commercial laying hens, understanding these properties is important for assuring safe locomotion. We tested the effects of wing area loss on mass, physiological cross-sectional area (PCSA), and estimated muscle stress (EMS) of the PECT and SUPRA in white-feathered laying hens. Treatments included Unclipped (*N* = 18), Half-Clipped with primaries removed (*N* = 18) and Fully-Clipped with the primaries and secondaries removed (*N* = 18). The mass and PCSA of the PECT and SUPRA did not vary significantly with treatment. Thus, laying hen muscle anatomy may be relatively resistant to changes in external wing morphology. We observed significant differences in EMS among treatments, as Unclipped birds exhibited the greatest EMS. This suggests that intact wings provide the greatest stimulus of external force for the primary flight muscles.

## 1. Introduction

Maintaining plumage quality is important for both wild and domesticated birds, as feather loss or damage can lead to reduced wing area [1] and impaired/reduced mobility [2]. Adult birds of many different species will generally undergo an annual or biannual moult in the wild to replace worn feathers [1]. During this period, feathers are shed, leaving some birds flightless. Subsequently, the flight muscles can atrophy due to disuse [3,4]. In domesticated chicken flocks kept for egg production (laying hens), loss of feathers can come from feather pecking and abrasions from housing equipment [5]. Feather pecking is present in up to 86% of commercial laying hen flocks, affecting all areas of the body, including the wing and tail feathers [5–7].

As ground birds (Galliformes), domestic chickens (*Gallus gallus domesticus)* rely heavily on bipedal walking and running and have limited flight abilities. Galliform birds are capable of flapping flight, but their flight is generally explosive, with greater wing beat frequency and larger power output requirements compared with similarly sized, non-Galliform flying counterparts (e.g. chukars [8] versus pigeons [9]). Any reduction in wing area, for example, through feather damage or loss, will increase wing loading (body mass per wing area) [10] and subsequently increase the required power output needed for flapping flight [11]. León *et al.* [12] showed that even fully wing-feathered chickens work at their maximum power output when performing flapping flight. While the flight feathers of the wings convert muscle power into aerodynamic power [13], the two main flight muscles, the pectoralis (PECT) and supracoracoideus (SUPRA), power avian wing movement [9]. The flight muscles can make up to 20% of an adult bird's total body mass [13]. The significantly larger PECT makes up approximately 17% of an adult bird's body mass and sits superficial to the smaller SUPRA, which only makes up about 2–4% of the body mass in comparison [13]. Both muscles originate on the keel and insert on the humerus, with the PECT inserting at the deltopectoral crest and the SUPRA inserting at the dorsal humeral head via a long central tendon that passes through the foramen triosseum [14]. During flapping flight the PECT powers the lift for weight support and thrust during the downstroke [9]. The SUPRA rotates, supinates and elevates the wing to overcome wing inertia but contributes much less to overall aerodynamic force than the PECT [9,14,15].

The PECT has a complex anatomy with some parallel but mostly bipennate fibres and a short tendon of insertion, whereas the SUPRA is bipennate with a long tendon of insertion [13]. Bipennate muscles have fascicles that attach to opposite sides of a central tendon [13]. The maximal muscle force these muscles can produce is proportional to the muscle's physiological cross-sectional area (PCSA) [3]. In addition, for a given volume, bipennate muscles have a greater PCSA and, therefore, can produce more force than parallel-fibred muscles [3]. Notably, muscle force exerted at the tendon of insertion is also affected by the pennation angle (*α*), the angle between the muscle fibre and the central tendon, with greater muscle stress required for a given force output as the angle increases [3,16,17]. The proportionally long tendon of insertion of the SUPRA is estimated to store and release elastic energy to assist with inertial work requirements for upstroke [9]. However, little is known about the architectural properties of the PECT and SUPRA of domestic chickens.

As a model for laying hens experiencing severe feather loss in commercial farming, we experimentally decreased wing area using controlled clipping of the primary and secondary flight feathers, which effectively reduced the use of elevated resources in aviaries [11]. Hens with all flight feathers left intact had lower descent velocities and descent angles compared with hens with clipped flight feathers, which is vital for slow, safe and controlled landings [12]. Additionally, wing-feather clipping reduced PECT thickness [18]. In this paper, we further explore the impact of wing-feather clipping on muscle mass, average fascicle length, pennation angle (*α*), PCSA and estimated muscle stress (EMS) in the PECT and SUPRA. First, we describe the architectural properties of the PECT and SUPRA, which are then used to calculate the PCSA, and used simplified models of aerodynamic and inertial forces to calculate EMS (kPa). Second, we use wing feather clipping to investigate the effects of wing area loss on the PECT and SUPRA muscle characteristics. We predicted atrophy of the muscles in the clipped groups, leading to reductions in mass and, potentially, increases in pennation angle that would in turn reduce PCSA and, for a given force requirement, increase EMS as a result of the reduction of elevated resource use/flapping flight capabilities [11].

## 2. Material and methods

### 2.1. Animals and housing

A total of 60 white domestic Lohmann LSL lite laying hens were housed in six aviary-style pens (183 cm L × 244 cm W × 290 cm H), with 10 hens per pen as part of a larger study assessing the impact of wing-feather clipping on potential keel bone injuries [18], changes in behaviour [11], muscle depth [18] and flight kinematics [12]. The floor was covered in 5 cm of wood shavings and each pen included two high platforms (122 cm L × 31 cm W) 70 cm above the ground on either side of the pen. In addition, one feeder was in the middle of the floor, and a second feeder secured to one of the two elevated platforms. Similarly, two nest-boxes were provided: one was placed against the back wall on the floor, and the second was secured to the second platform. A high perch (5 cm diameter) placed at a height of 150 cm spanning the width of the pen was installed near the back wall. Automatic nipple drinkers were provided. The pens were kept at 21°C and had a 14 : 10 h light–dark cycle and a 30-minute dawn/dusk period.

### 2.2. Wing-feather clipping treatment

**Figure 1.**
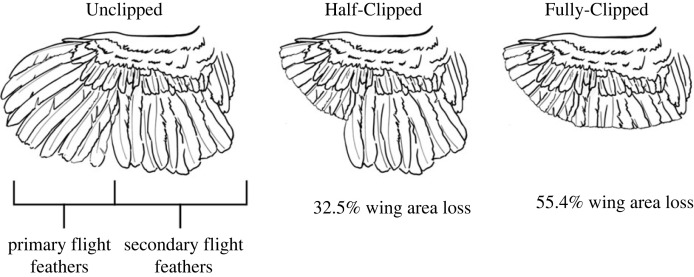
Diagrammatic representation of the wing-feather clipping treatments. Unclipped, where no flight feathers were clipped; Half-Clipped, where the 10 primary flight feathers on both wings were clipped; Fully-Clipped where all primary and secondary flight feathers on both wings were clipped along the coverts.

Wing-feather clipping treatments were applied as described by Garant *et al.* [11] at 34 weeks of age during the fall season. Three birds from each pen were randomly assigned to one of three treatment groups (figure 1): Unclipped, where all flight feathers were left intact (table 1; wing loading 162.4 ± 11.5 kg m^−2^); Half-Clipped, where the 10 primary flight feathers were clipped symmetrically across both wings (32.5% wing area loss and 249.1 ± 29.1 kg m^−2^ wing loading), and Fully-Clipped where all secondary and primary flight feathers were clipped symmetrically across both wings (55.4% wing area loss and 378.9 ± 53.9 kg m^−2^ wing loading). The primary and secondary covert feathers were used as a guideline when clipping the feathers using scissors.

**Table 1.** Morphometrics of experimentally clipped wings of white layer hens (*Gallus gallus domesticus*). Values represent means ± s.d. for one wing extended as in mid-downstroke.

length (m)	0.3 ± 0.02	0.2 ± 0.02	0.2 ± 0.02
area (m^2^)	0.05 ± 0.003	0.03 ± 0.002	0.02 ± 0.002
second moment of area (m^4^ * 10^−5^)	11.7 ± 1.90	3.4 ± 0.70	2.2 ± 0.60

### 2.3. Dissection and muscle collection

All hens were euthanized using CO_2_ by trained personnel at 42 weeks of age (eight weeks after the clipping treatment) and kept frozen at −18°C until further analysis. From this population, a randomly selected subsample of 18 birds (6 hens/clipping treatment) was dissected to assess PCSA and calculate EMS. Dissections took place over 3 days in the winter, with approximately eight hens dissected per day by two trained researchers (trained simultaneously using the same protocol). The same researcher collected all measurements on one hen. Hens from each treatment were dissected on each day and equally divided among the researchers who were blinded to the treatments. Carcasses were left to thaw for an average of 9.5 h at room temperature. Hens that were not immediately dissected were kept in the fridge at 1°C until dissection. Each dissection lasted, on average 2 h, and all carcasses were dissected within 6 h of thawing.

Dissections were carried out by following the methods of Casey-Trott *et al.* [19]. In brief, whole carcasses (including feathers and viscera) were weighed immediately prior to dissection by placing the carcass in a reusable bag and using a luggage scale (Maple Leaf Travel Accessories, ACI Brands, Inc., Ontario, Canada). Muscles were removed in the order of left PECT, left SUPRA, right PECT and right SUPRA. Visible fat and connective tissue were removed as best as possible. Immediately after removal, muscles were weighed on an analytical balance (Mettler Toledo AE200 Analytical Balance, Mettler Toledo, Ontario, Canada) and placed on a piece of cardstock in preparation for measuring fascicle length and pennation angle.

### 2.4. Muscle measurements

**Figure 2.**
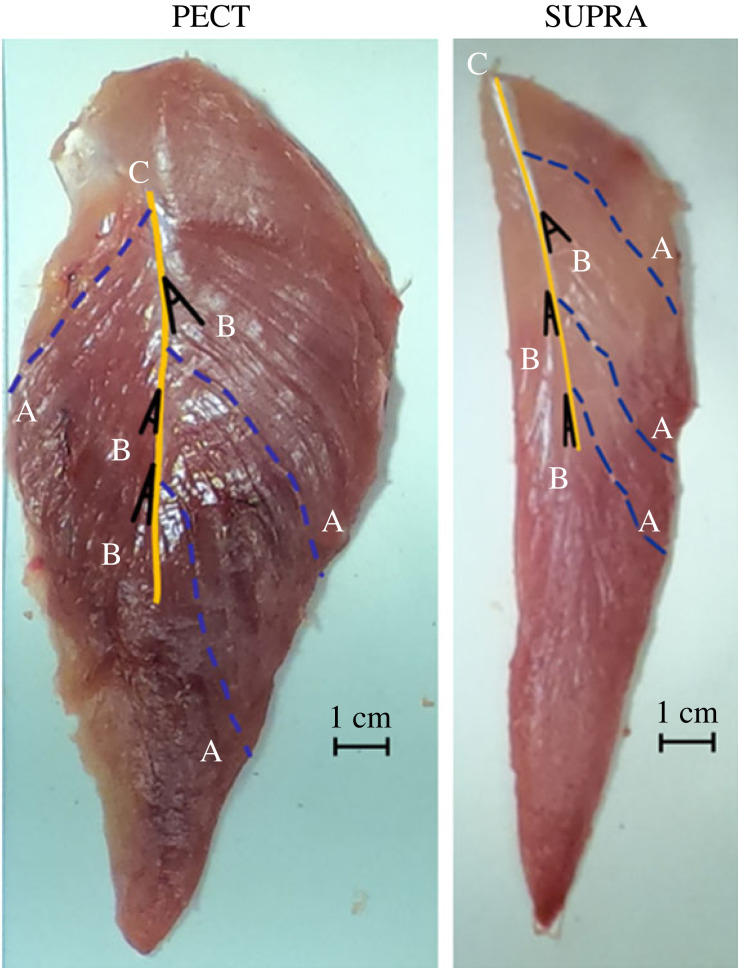
The deep side of the pectoralis (PECT) and supracoracoideus (SUPRA). (*a*) Dashed blue lines are examples of muscle fascicles measured in cm. (*b*) Solid black lines indicate pennation angles measured in degrees between two intersecting lines (orientation of the fascicle and central tendon). (*c*) The central tendon is indicated by the solid yellow line.

The fascicle length and pennation angles (figure 2) of each muscle were measured. Ten muscle fascicles were chosen randomly on both the superficial and deep sides of each muscle (total of 20 measurements). A flexible tape measure was used to follow the curves of each muscle fibre to measure fibre length (cm). Ten pennation angles were chosen at random and measured using an electronic protractor (Beslands 0–200 mm/8 inches Digital Protractor, Beilong Tool, Zhejiang, China) on both the superficial and deep sides of each muscle (total 20 measurements) in degrees (°). Pennation angles were measured by lining up the base/reference line of the protractor with the central tendon (figure 2). For the PECT, one of the 10 pennation angles was assumed to be 0° as a small portion of the PECT does not directly insert into the central tendon.

### 2.5. Physiological cross-sectional area and estimated muscle stress calculations

Muscle fascicle length was converted from cm to m, and the pennation angles were converted from degrees to radians prior to their use in calculating the PCSA and EMS. The PCSA was calculated by dividing the mass of the muscle (*M*, in kg) by the product of muscle density (assumed 1060 kg m^−3^; [20]) and average fascicle length (*L*, in m) of either PECT or SUPRA.

Equation (2.1). Calculation for the PCSA (in m^2^) where *M* is muscle mass (in kg), *p* is muscle density (1060 kg m^−3^; [20]), and *L* is average fascicle length (*L*, in m).

2.1 $$\mathrm{PCSA}=\frac{M}{\;pL}.$$

### 2.6. Estimated muscle stress

We developed simplified models of aerodynamic and inertial forces to estimate muscle stress for the PECT and SUPRA, respectively. We assumed power for weight support during downstroke to be the primary role of the PECT and inertial power was the primary role of the SUPRA. Data from León *et al.* [12] was used for relevant whole-body and wing kinematics, as these were from the same hens and treatments used in the current study. Induced power for vertical weight support dominates requirements for slow flight [21,22]; therefore, we used a flapping-wing approximation of the Rankine–Froude momentum theory of propellers [23] and reasoned that average vertical force in a momentum jet must equal body mass multiplied by the vertical acceleration reported in León *et al.* [12], where proportion of weight supported was 0.73, 0.53 and 0.79 for Unclipped, Half-Clipped and Fully-Clipped birds, respectively. Each PECT provides force to support half body weight, therefore, body weight (N) was divided in half and multiplied by the proportion of weight supported in slow descending flight. As the downstroke provides most or all of the weight support during slow flight in all birds except hummingbirds [24], we then divided by 0.52, the proportion of the wingbeat cycle consisting of downstroke as calculated from data in León *et al.* [12]. To solve the average force balance for downstroke, the centre of pressure of the wing was assumed to be located at 62.5% of the length of the wing [25]. Aerodynamic moment (Nm) was calculated as the body weight supported by one wing during downstroke multiplied by the spanwise distance to the centre of pressure of the wing, assumed from empirical study [25] to be 0.625 multiplied by the wing length (table 1). Muscle force was then found by dividing the aerodynamic moment by the average moment arm of the hen PECT attaching to the deltopectoral crest of the humerus, which was measured from three birds (0.0176 ± 0.0013 m). We then used this average value to calculate EMS (see equation (2.2), below).

The upstroke in chickens and other Galliform birds is understood to be largely aerodynamically inactive such that muscle force is required only to accelerate the wing and overcome wing inertia during the first half of upstroke [9,21,24,26]. The torque required to accelerate the wing should equal the moment of inertia of the wing (*I*; kg m^2^) multiplied by its angular acceleration (*θ*; rad s^−2^; [27]. We measured *I* for one hen in each treatment using standard, spanwise strip measurements [27,28]: Unclipped = 6.8 × 10^−4^ kg m^2^, Half-Clipped = 4.2 × 10^−4^ kg m^2^ and Fully-Clipped = 3.3 × 10^−4^ kg m^2^. The wing was stretched to have a straight leading edge emulating the posture at mid-downstroke. The wing was then cut into spanwise strips 2 cm in width, and the mass of each strip (including all muscle, bone and feathers) was measured using an analytical balance (Analytical Balance ME104E, Mettler Toledo, Ontario, Canada). Our calculation assumed point masses per strip [27]. Chickens flex their wings using a ‘tip reversal’ upstroke, as in other Galliform birds [8]. Time-resolved three-dimensional kinematics are necessary for estimating instantaneous changes in inertial force requirements. Lacking data with such resolution, we instead measured span ratio (mid-upstroke wrist span divided by mid-downstroke wrist span) using the kinematics results in León *et al.* [12] at 0.625. Each individual segment distance from the shoulder was then multiplied by this span ratio to calculate *I*. Angular velocity of the upstroke was measured as wingbeat amplitude (rad) divided by upstroke duration [12]. Treating changes in wing velocity as a sinusoidal function, we calculated the peak angular velocity at mid-upstroke as average angular velocity during the upstroke divided by 0.64. Angular acceleration (*θ*) during the first half of upstroke was then changed in angular velocity from 0 rad s^−1^ at the start of upstroke to the peak angular velocity at mid-stroke, divided by the duration of the first half of upstroke. Dividing *Iθ* by the moment arm of the SUPRA operating about the shoulder, taken to be 0.0052 ± 0.00008 m (average taken from measurements of three birds), yielded the average SUPRA force applied to a wing during the first half of the upstroke. During the second half of the upstroke, we assumed force was dissipated either as a pulse of thrust [29] or as energy absorbed during active lengthening in the pectoralis [9,26]. As in the PECT, we then calculated EMS using equation (2.2).

Equation (2.2). Calculation for EMS (kPa). Where *F* (N) is muscle force calculated according to the specific muscle (PECT or SUPRA, detailed above), PCSA is the physiological cross-sectional area (m^2^) from equation (2.1), and *α* is average pennation angle (radians).

2.2 $$\mathrm{EMS}=\frac{F}{\mathrm{PCSA}\times \cos(\alpha )}.$$

### 2.7. Statistical methods

For ease of communication herein, we converted units of PCSA from m^2^ to cm^2^ and EMS from Pa to kPa. Left- and right-side muscle data, as well as superficial and deep-side data were averaged for each outcome variable as no significant differences were found after using generalized mixed model procedures (PROC GLIMMIX) in SAS OnDemand for Academics SAS Studio v. 9.04 (2021, SAS Institute Inc., Cary, NC, USA) to analyse statistical differences between said sides (*p* > 0.05, data not shown).

PECT and SUPRA data were analysed separately. Three outcome variables were assessed as part of the analysis: muscle mass (g), PCSA (cm^2^) and EMS (kPa) for each muscle. PROC GLIMMIX was used in SAS OnDemand for Academics: SAS Studio v. 9.04 (2021, SAS Institute Inc., Cary, NC, USA) to perform statistical analysis on all outcome variables to determine significant differences between clipping groups. The wing-feather clipping status (three levels: Unclipped, Half-Clipped, Fully-Clipped) was included as the fixed effect for all three outcome variables. The hen's body weight (kg) was added as a covariate for muscle mass and PCSA, but not for EMS, as body mass is accounted for when calculating EMS.

Studentized residual plots were used to assess normality of the data and determine the best distribution that fit the data. Therefore, to meet normality assumptions, the model for PCSA was kept as a Gaussian distribution, while the models for muscle mass and EMS used the lognormal distribution for both PECT and SUPRA data. Descriptive statistics are presented with the standard deviation (s.d.). Results from the statistical analysis are presented as least-squared means (LSM) or as back-transformed LSM with the standard error (s.e.). A Tukey-Kramer *p*-value adjustment was used to assess multiple comparisons, the *α* determination of significance was 0.05.

## 3. Results

**Figure 3.**
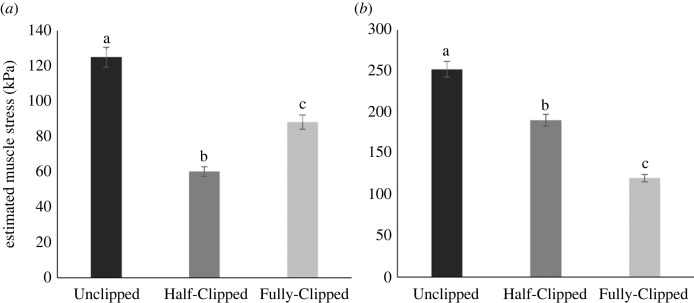
LSM ± standard error (s.e.) of the estimated muscle stress (EMS, kPa) of (*a*) the pectoralis (PECT) and (*b*) the supracoracoideus (SUPRA) in white-feathered laying hens across three different wing-feather clipping treatments (Unclipped, Half-Clipped and Fully-Clipped). Different letters denote significant differences (*p* < 0.05).

Muscle mass, PCSA and EMS for both the PECT and SUPRA followed similar patterns where they were largest in the Unclipped group and smallest in the Half-Clipped group, except the EMS of the SUPRA as it was smallest in the Fully-Clipped group (figure 3, table 2).

**Table 2.** Morphological data for the pectoralis (PECT) and supracoracoideus (SUPRA) muscles of white-feathered laying hens across the three wing-feather loss/clipping groups (Unclipped, Half-Clipped, Fully-Clipped, *n* = 6 birds per treatment). This includes mean ± standard deviation (s.d.), minimum and maximum values of body weight (g), muscle fascicle length (cm), pennation angle (*α*, radians), muscle mass (g), physiological cross-sectional area (PCSA, cm^2^) and estimated muscle stress (EMS, kPa).

variable	Unclipped			Half-Clipped			Fully-Clipped		
	mean ± s.d.	min	max	mean ± s.d.	min	max	mean ± s.d.	min	max
body weight (g)	1910.0 ± 0.14	1770.0	2170.0	1648.3 ± 0.16	1380.0	1780.0	1675.0 ± 0.17	1370.0	1880.0
PECT									
mass (g)	77.5 ± 6.55	66.3	86.6	68.0 ± 5.72	61.6	77.0	68.4 ± 6.69	62.1	79.8
fascicle length (cm)	6.3 ± 0.41	5.9	6.9	6.5 ± 0.28	6.1	6.8	6.6 ± 0.43	5.7	6.9
pennation angle (radians)	0.5 ± 0.08	0.4	0.6	0.5 ± 0.06	0.4	0.6	0.5 ± 0.07	0.4	0.6
PCSA (cm^2^)	11.5 ± 1.22	9.4	13.1	10.0 ± 0.72	9.1	11.1	10.1 ± 0.92	9.3	11.9
EMS (kPa)	125.5 ± 12.86	109.8	146.2	60.5 ± 6.63	53.7	69.6	88.7 ± 11.72	79.3	111.6
SUPRA									
mass (g)	26.5 ± 1.53	24.2	28.7	24.3 ± 1.51	22.8	26.9	25.5 ± 2.20	22.8	29.0
fascicle length (cm)	3.6 ± 0.32	3.0	3.9	3.8 ± 0.19	3.5	4.1	3.8 ± 0.25	3.5	4.1
pennation angle (radians)	0.5 ± 0.04	0.5	0.6	0.5 ± 0.08	0.4	0.6	0.6 ± 0.06	0.5	0.6
PCSA (cm^2^)	6.8 ± 0.62	6.1	7.9	6.2 ± 0.37	5.5	6.6	6.2 ± 0.70	5.3	7.0
EMS (kPa)	253.3 ± 27.30	202.5	274.2	190.6 ± 14.75	175.4	212.0	120.3 ± 9.81	108.9	136.2

Overall, wing area loss (through wing-feather clipping) did not significantly affect the muscle mass of either the PECT (73.6 ± 2.79 g (Unclipped) versus 69.5 ± 2.28 g (Half-Clipped) versus 69.7 ± 2.37 g (Fully-Clipped); *F*_2,14_ = 0.63, *p* = 0.5454) or SUPRA muscles (25.5 ± 0.77 g (Unclipped) versus 24.74 ± 0.67 g (Half-Clipped) versus 25.8 ± 0.68 g (Fully-Clipped); *F*_2,14_ = 0.67, *p* = 0.5258). There was no significant impact of wing area loss on the PCSA of either muscle (PECT: 11.6 ± 0.33 cm^2^ (Unclipped) versus 10.2 ± 0.28 cm^2^ (Half-Clipped) versus 10.3 ± 0.28 cm^2^ (Fully-Clipped), *F*_2,14_ = 1.32, *p* = 0.2978; SUPRA: 6.6 ± 0.31 cm^2^ (Unclipped) versus 6.3 ± 0.28 cm^2^ (Half-Clipped) versus 6.3 ± 0.28 cm^2^ (Fully-Clipped), *F*_2,14_ = 0.95, *p* = 0.4097).

However, wing-feather clipping inducing wing area loss did significantly affect the EMS of the PECT (*F*_2,15_ = 64.90, *p* < 0.05) and SUPRA (*F*_2,15_ = 98.37, *p* < 0.05), where EMS in the PECT was highest in the Unclipped birds, followed by Fully-Clipped birds and lowest in the Half-Clipped birds (figure 3*a*). Similarly, for the SUPRA, EMS was also highest in the Unclipped birds; however, it was intermediate in Half-Clipped birds and least in the Fully-Clipped birds (figure 3*b*). EMS of the SUPRA was approximately double that of the PECT (figure 3).

## 4. Discussion

Wing-feather clipping treatments resulting in wing area loss applied to the laying hens did not significantly affect muscle mass or PCSA of the PECT or the SUPRA. The initial hypothesis predicted that the Half-Clipped (32.55% wing area loss) and Fully-Clipped (55.4% wing area loss) groups would have lighter and smaller muscles than the Unclipped group, as wing area loss would hinder flapping flight and discourage the use of the PECT and SUPRA. This lack of difference in muscle mass and PCSA is surprising as the wing-feather clipping treatment drastically reduced wing area by up to 55% per wing [11], resulting in a significant decline in elevated resource usage [11] and an approximate 6% decrease in PECT muscle thickness when ultrasound was used to measure effects in live birds six weeks after clipping [18]. Therefore, we hypothesized that decreases in muscle mass and PCSA would be reasonably observable eight weeks post-clipping in the present study. In wild waterfowl such as geese and grebes, muscle atrophy is commonly observed within a single moulting period, which leaves them flightless for several weeks (approx. four weeks) [3,30]. Additionally, other studies have shown that migratory birds such as red knots (*Calidris canutus*) see both increases and subsequent decreases in pectoralis thickness in less than 12 weeks [31]. By contrast, decreases in muscle mass in the present study were not seen in eight weeks, let alone recovery of the muscle mass. However, the present study suggests that additional time may be needed to see detectable differences in muscle mass and PCSA, specifically in the domestic laying hen, or perhaps that domestic laying hen muscle architecture is resistant to change in comparison with wild species of waterfowl and migratory species. A small sample size also limits the present study, and future studies would benefit from exploring the effects of flight feather clipping on the PECT and SUPRA in a larger population.

Although decreases in PCSA due to wing area reduction were not seen, wing-feather clipping and subsequent effects on flight capability were still found to significantly affect the EMS of both the PECT and SUPRA. The EMS in this study was estimated using the amount of aerodynamic force and inertial torque the PECT and SUPRA would need to match, respectively. Therefore, EMS herein is an integration of differing amounts of wing length, area, wing second moment of area, wing moment of inertia and exhibited flight behaviour (table 1; [12]). Taken together with the fact that PCSA did not differ significantly according to treatment, EMS in this study is more a reflection of behaviour in flight (vertical support of body weight) for PECT EMS and largely invariant angular velocities of the wing coupled with large changes in moment of inertia (table 2). Large EMS may represent normal feedback that elicits flight behaviour in hens, as previous work indicates that when the wing area is significantly reduced (up to 55% wing area reduction per wing) in these birds, they may opt for other methods of locomotion, such as ground locomotion [11,18]. Garant *et al.* [11] showed that elevated resource use/aerial locomotion is decreased by 42%, and leg muscle thickness is increased following wing-feather clipping [11,18]. Furthermore, León *et al.* [12] demonstrated that laying hens do not adjust their wing kinematics to adapt to experimentally reduced wing area as in the present study, suggesting that the birds with intact wings are already at their maximum capacity for aerodynamic power output. Therefore, unlike wild and more volant species of birds such as the pied flycatchers (*Ficedula hypoleuca*) and rock pigeons (*Columba livia*) that are able to modulate their flight in response to feather loss by flying faster or increasing flapping frequency to maintain flight performance [32,33], domesticated laying hens may not have the same flexibility to accommodate loss of wing area. This may then pose problems for welfare in multi-tiered systems as these birds would have difficulty in reaching elevated resources [11].

The PECT of the Half-Clipped birds were estimated to produce significantly less EMS than both the Unclipped and Fully-Clipped groups. Previous work in flight kinematics using these birds showed that the Half-Clipped birds were not as adept at supporting their own body weight in comparison with the Fully-Clipped group despite the Half-Clipped group having a larger wing area [12]. While the Unclipped and Fully-Clipped groups could support around 76% of their body weight, the Half-Clipped birds could only support 47% of their body weight. Additionally, they had the highest vertical acceleration during descent [12]. We hypothesize that the relatively poor performance was influenced by the shape of the wings left by the Half-Clipped clipping treatment, as the shape of the wing is important in flight stabilization [13]. Although the Fully-Clipped group had the highest wing area reduction, the shape left by clipping both the primary and secondary flight feathers still mimicked the shape of a fully intact wing, as there was generally the same amount of area distributed throughout the length of the wing. However, as only the primary flight feathers were cut in the Half-Clipped group, these birds were left with a wing shape where there was a large wing area close to the body contrasted with a large area missing from the outer part of the wing. Nonetheless, the effect of wing shape in this context needs to be studied further.

The laying hen is a relatively large bird with primarily fast-twitch glycolytic fibres in its PECT [34]. In related birds, the PECT muscle strain increases with increasing body size to maintain flight capabilities [8]. Thus, it is not surprising that EMS in the PECT and SUPRA of laying hen was greater (figure 3) than that reported for other species, such as for the PECT in pigeons (50–58 kPa; [9]) and starlings (34 kPa; [35]) and the SUPRA in pigeons (85–125 kPa; [9]) for which *in vivo* measures of muscle stress and strain are reported. Muscle force (*F*) was estimated in this study using the moment arm of each muscle and average aerodynamic force (PECT) or inertial force (SUPRA), our estimates could be improved in future studies by using *in vivo* measures, specifically electromyography (EMG) to measure neuromuscular activity and sonomicrometry to measure muscle strain (e.g. Tobalske & Biewener [9]). Galliform birds, including chickens, have a deltopectoral crest with a shape that precludes *in vivo* measures of muscle stress [8]. However, new methods using an aerodynamic force platform and time-resolved measures of three-dimensional wing shape could be used to calculate muscle force [26]. In addition, Iyer *et al.* [36] present two potential methods of measuring muscle contractility and force *in vivo* and *in situ*, respectively, by measuring muscle torque around a joint and muscle tension by attaching a tendon to a load cell. Mechanomyography (MMG) is yet another potential method for future studies that measure muscle vibration and stiffness produced during contraction, which can be related back to muscle force [37,38].

## 5. Conclusion

Wing area loss significantly affected the EMS of the PECT and SUPRA. EMS of the PECT was smallest in the Half-Clipped group despite the Fully-Clipped group having less than 50% of their wing area left intact which may be due to changes in wing shape and/or unmeasured alteration of neuromuscular coordination. Loss of wing area did not significantly affect the muscle mass or PCSA of the PECT or SUPRA in domestic laying hens, despite previous studies showing loss of wing area affects their ability to access desirable elevated resources. It is possible that laying hen muscle physiology is such that mass and PCSA are more resistant to change than in wild and more volant species. Rather than increasing muscle size to compensate for increases in aerodynamic demand due to reduction in wing area, laying hens may have instead chosen alternative methods of terrestrial locomotion.

## Data Availability

Raw data has been made publicly available. The data are provided in electronic supplementary material [39].
